# Supplementary material for: Educational Inequalities in COVID-19 Vaccination: A Cross-Sectional Study of the Adult Population in the Lazio Region, Italy
Source: Vaccines (Basel). 2022 Feb 25;10(3):364. doi: 10.3390/vaccines10030364 (PMC8950687; doi:10.3390/vaccines10030364)
Supplement: Supplementary file 1 [file vaccines-10-00364-s001.zip › Table S1.pdf]

Table S1. Characteristics of the study population included and not included in the study according to vaccination status on 22nd December 2021.

	Included subjects					Not-included subjects				
	N	%	% Vaccination in the study population			N	%	% Vaccination in subjects excluded from the study		
			% No vaccination	% Partial vaccination	% Completed vaccination			% No vaccination	% Partial vaccination	% Completed vaccination
**Total**	3,186,728	100	10.3	1.5	88.1	438,049	100	27.1	2.5	70.4
**Sex**										
Men	1,467,434	46.0	10.0	1.5	88.4	206,793	47.2	26.9	2.5	70.5
Women	1,719,294	54.0	10.6	1.5	87.9	231,256	52.8	27.3	2.4	70.2
**Age, mean (sd)**	58.9 (14.3)		56.2 (14.8)	53.8 (13.9)	59.3 (14.2)	52.7 (13.9)		52.9 (14.2)	48.7 (11.8)	52.7 (13.8)
**Place of birth**										
Italy	2,931,917	92.0	9.0	1.5	89.5	273,981	62.5	19.6	2.5	77.9
Other Countries	254,811	8.0	25.4	2.1	72.5	164,068	37.5	39.7	2.5	57.8
**Number of chronic conditions**										
0	1,597,725	50.1	13.0	1.9	85.1	300,042	68.5	31.6	2.7	65.7
1	738,096	23.2	8.5	1.3	90.2	73,356	16.7	19.1	2.3	78.6
2	403,689	12.7	7.1	1.1	91.8	32,316	7.4	15.9	1.9	82.3
3+	447,218	14.0	6.7	1.0	92.4	32,335	7.4	14.9	1.6	83.5
**Deprivation quantile**										
Q1	647,691	20.3	9.2	1.3	89.5	92,302	21.1	26.2	2.3	71.5
Q2	642,586	20.2	9.7	1.4	88.9	85,458	19.5	25.6	2.4	72.0
Q3	637,238	20.0	10.0	1.5	88.5	80,184	18.3	25.5	2.3	72.2
Q4	627,070	19.7	10.7	1.6	87.8	81,555	18.6	27.0	2.6	70.4
Q5	616,831	19.4	11.9	1.8	86.4	91,793	21.0	28.4	2.8	68.8
m.i.	15,312	0.5	29.5	1.7	68.8	6,757	1.5	64.3	2.1	33.6
