# Supplementary figures and images for: Educational Inequalities in COVID-19 Vaccination: A Cross-Sectional Study of the Adult Population in the Lazio Region, Italy
Source: Vaccines (Basel). 2022 Feb 25;10(3):364. doi: 10.3390/vaccines10030364 (PMC8950687; doi:10.3390/vaccines10030364)

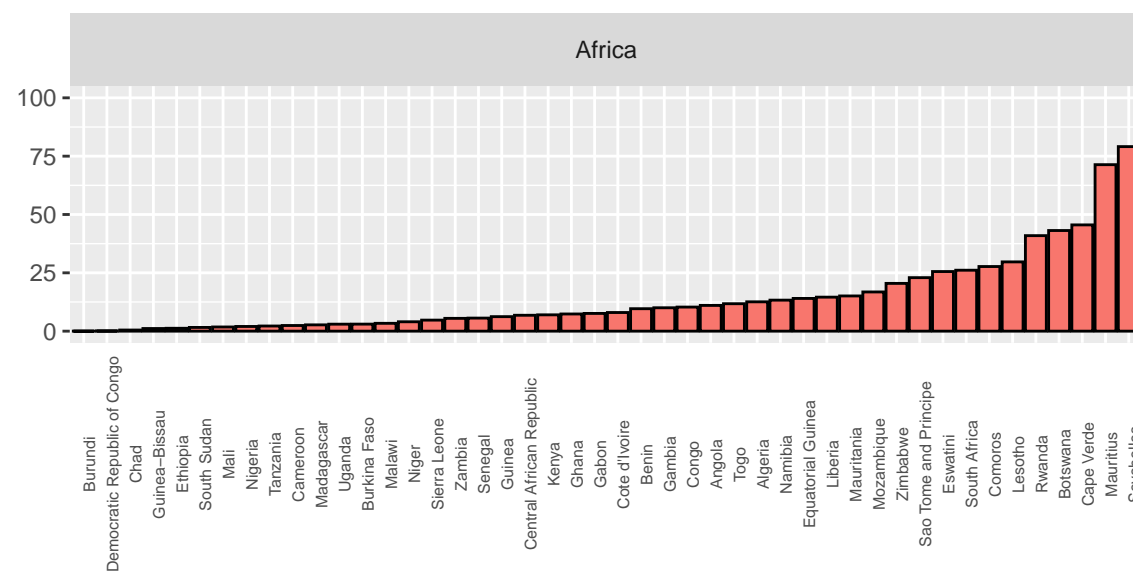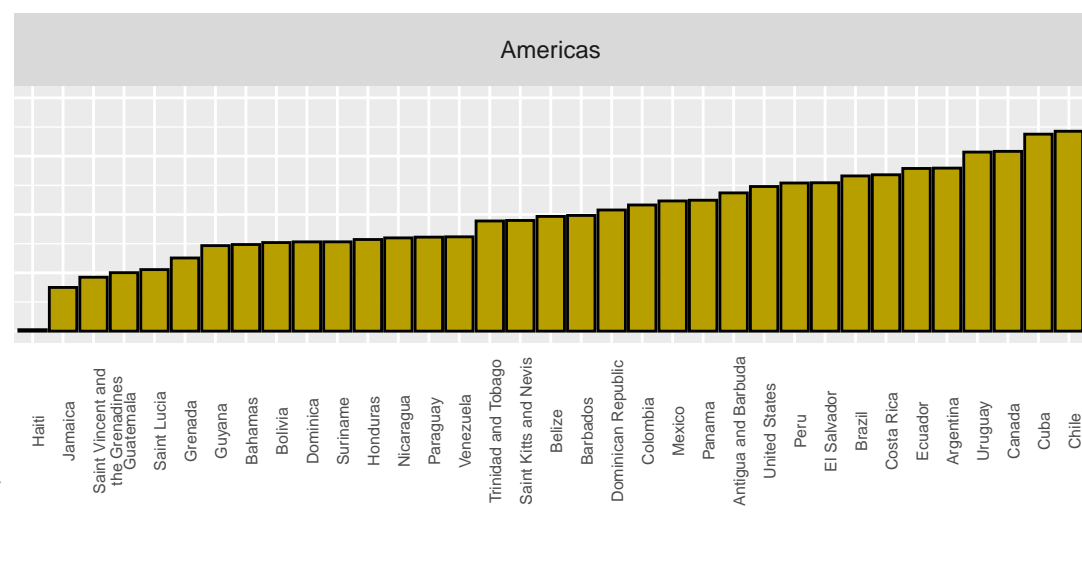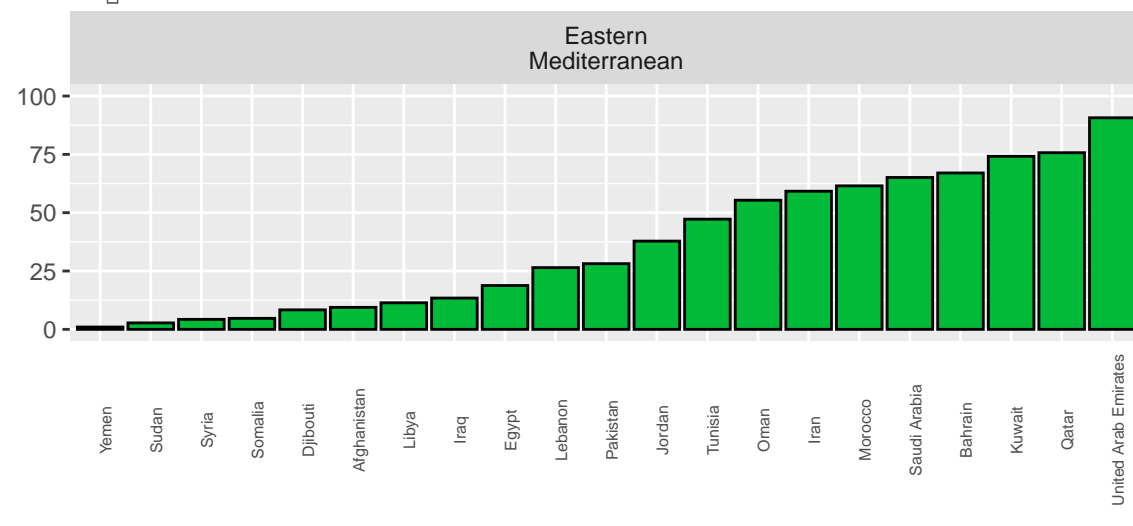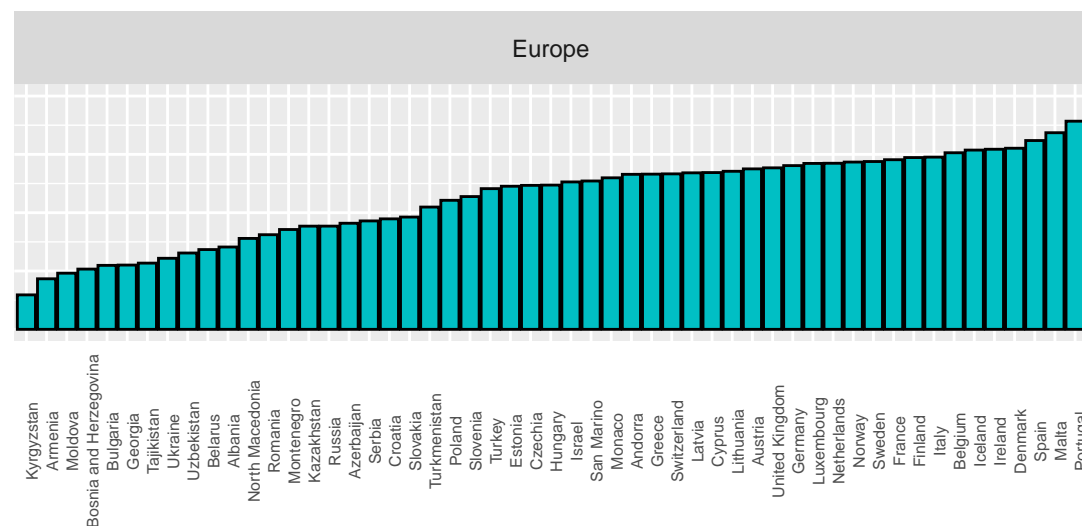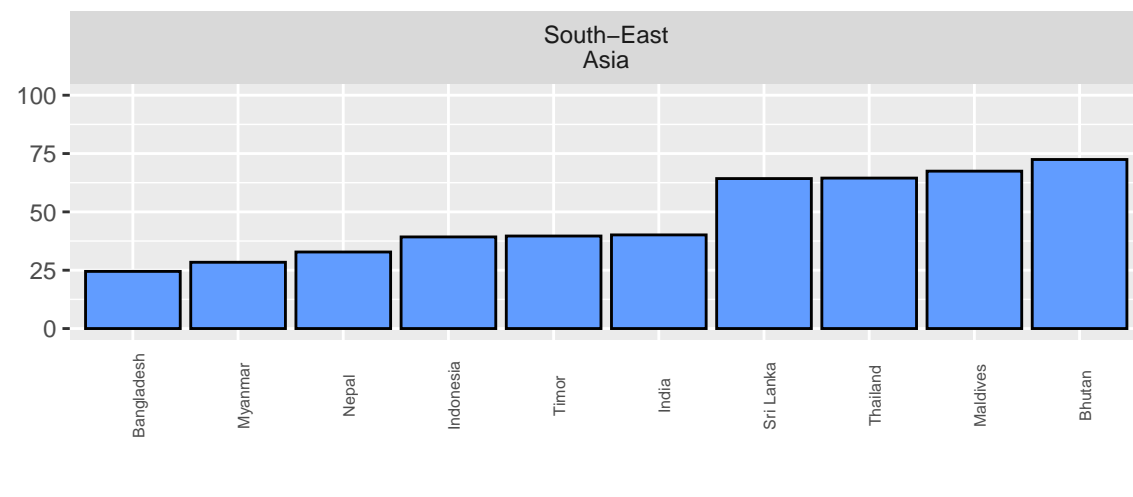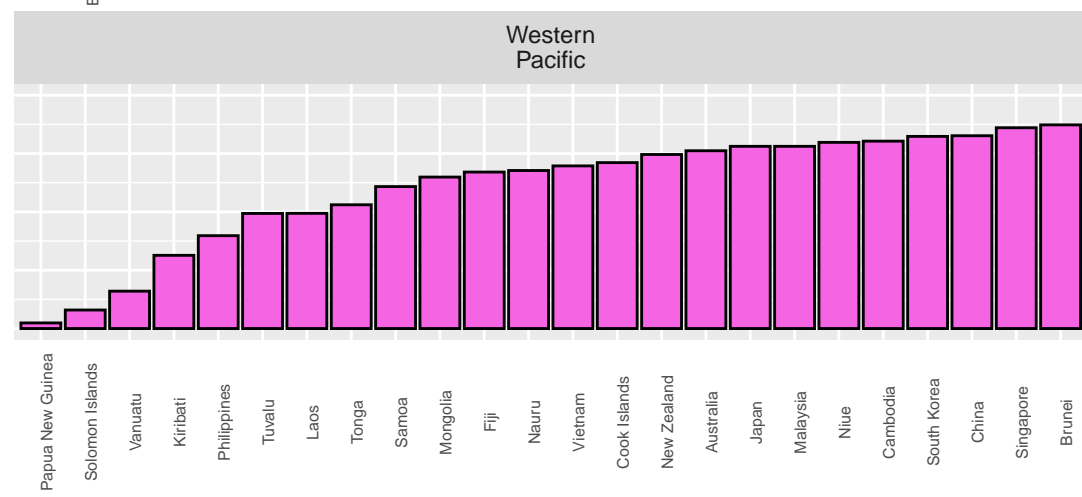

Supplement: Supplementary file 1 [file vaccines-10-00364-s001.zip › Figure S1.pdf]
